# Antiatherosclerotic Effect and Molecular Mechanism of Salidroside

**DOI:** 10.31083/j.rcm2404097

**Published:** 2023-03-23

**Authors:** Si-Fan Fei, De-Bing Tong, Fang Jia

**Affiliations:** ^1^Department of Cardiovascular Medicine, The First People's Hospital of Changzhou, The Third Affiliated Hospital of Soochow University, 213000 Changzhou, Jiangsu, China

**Keywords:** salidroside, atherosclerosis, endothelial dysfunction, cell targets, lipid metabolism, gut microbiota

## Abstract

Atherosclerotic cardiovascular disease is currently the leading cause of death worldwide. Its pathophysiological basis includes endothelial dysfunction, macrophage activation, vascular smooth muscle cell (VSMC) proliferation, lipid metabolism, platelet aggregation, and changes in the gut microbiota. Salidroside has beneficial effects on atherosclerosis through multiple pathways. In this review, we present studies on the regulatory effect of salidroside on atherosclerosis. Furthermore, we report the protective effects of salidroside against atherosclerosis by ameliorating endothelial dysfunction, suppressing macrophage activation and polarization, inhibiting VSMC proliferation, adjusting lipid metabolism, attenuating platelet aggregation, and modulating the gut microbiota. This review provides further understanding of the molecular mechanism of salidroside and new ideas for atherosclerosis management.

## 1. Introduction

**Fig. 1.**
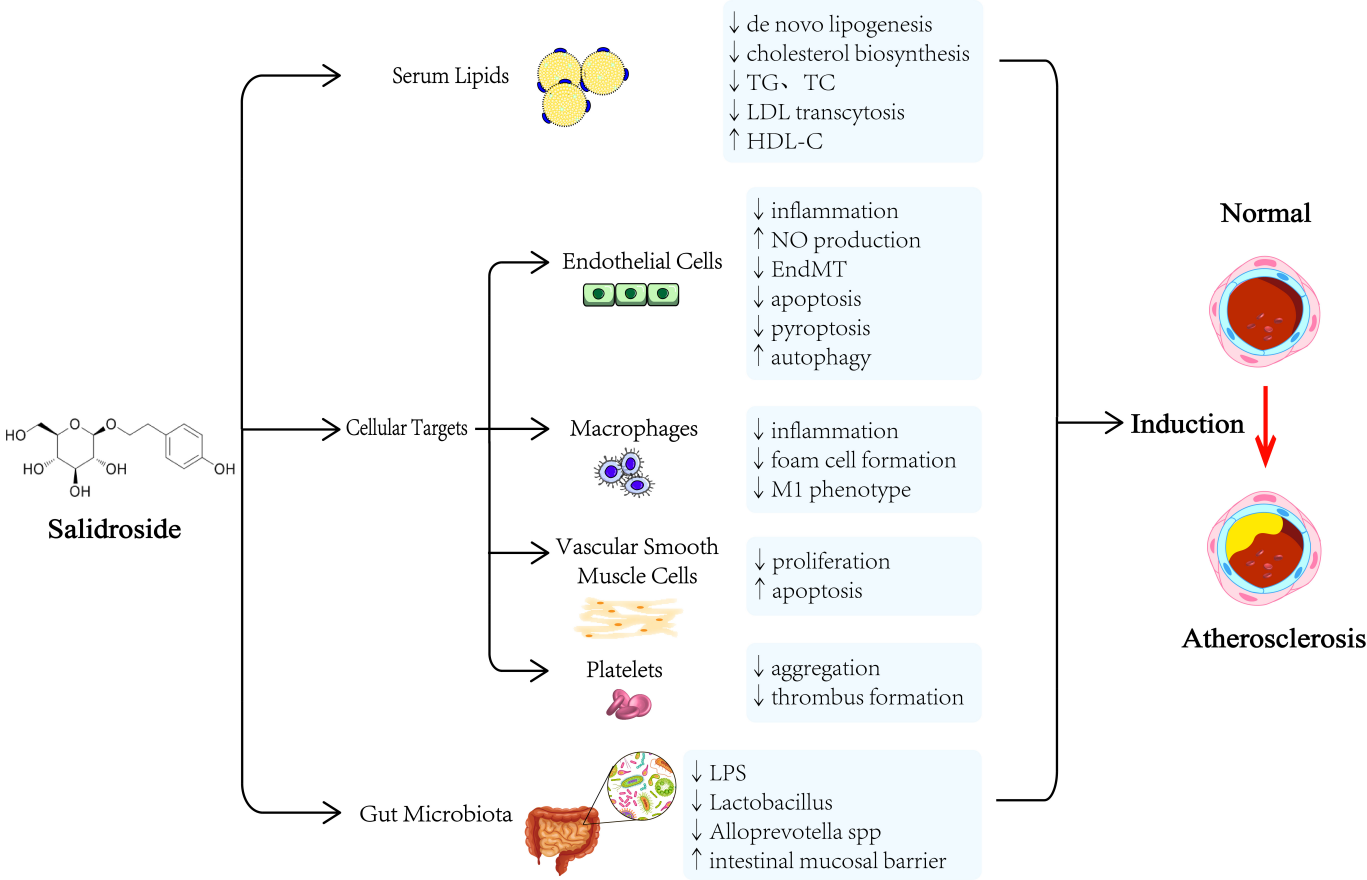
**The underlying mechanisms of salidroside in protecting against atherosclerosis**. TC, total cholesterol; TG, triglyceride (TG); LDL, low-density lipoprotein; NO, nitric oxide; HDL-C, high-density lipoprotein cholesterol; EndMT, endothelial-mesenchymal transition; LPS, lipopolysaccharide.

With the aging of the population, the morbidity and mortality of cardiovascular disease (CVD) remain high. In China, the number of deaths due to CVD was nearly 3.97 million, and the prevalence of CVD was estimated to be 93.8 million in 2016 [1]. The common pathological basis of CVD is atherosclerosis. The complex pathophysiologic process of atherosclerosis includes dyslipidemia, oxidative stress, endothelial dysfunction, thrombocyte aggregation [2, 3], foam cell formation and accumulation [4], and vascular smooth muscle cell (VSMC) migration and proliferation [5]. Oxidative modification and subendothelial retention of low-density lipoprotein cholesterol (LDL-C) represent the initial events in atherogenesis [6]. Oxidized low-density lipoprotein (Ox-LDL) enters the intima-media of the vascular wall and contributes to atherosclerotic plaque formation and progression by inducing endothelial cell (EC) activation and dysfunction, macrophage foam cell formation, and vascular smooth muscle cell (VSMC) migration and proliferation [7]. At present, statins [8] and antiplatelet therapy [9] are widely used to prevent atherosclerosis-related complications, and the effect of these therapies has pros and cons. For example, statins may lead to hepatotoxicity and skeletal muscle toxicity [10], and antiplatelet therapy inevitably leads to a risk of hemorrhage [11]. In recent years, interest in the role of herbal plants in treating CVD has grown. Chinese herbal medicine has long been used for the treatment of atherosclerotic complications [12]. Many studies suggest that salidroside, which has low toxicity and few side effects, possesses a wide range of biological properties, such as inhibiting inflammation, regulating dyslipidemia, improving endothelial function [13, 14, 15], suppressing macrophage phenotypic switching, decreasing the proliferation of VSMCs and impairing the activation of platelets. Thus, salidroside may be a valuable and promising drug candidate for the treatment of CVDs, but it is not in widespread use in clinical practice. In particular, salidroside can influence the gut microbiota; however, the mechanism that drives this phenomenon remains unclear. In this review, we provide an overview of the molecular mechanism by which salidroside attenuates atherosclerosis. The underlying mechanisms of salidroside in protecting against atherosclerosis as shown in Fig. 1.

## 2. Effects of Salidroside on Ameliorating Endothelial Dysfunction

Considerable evidence has suggested that dysfunction of the vascular endothelium plays a significant role in atherosclerosis development and progression. First, excessive reactive oxygen species (ROS) [16] and malondialdehyde (MDA) [17] can increase oxidative stress, which is linked to endothelial dysfunction and atherogenesis. Second, due to decreases in nitric oxide (NO) and endothelial nitric oxide synthase (eNOS) levels, endothelium-dependent vasodilation is impaired, which confers a risk of atherogenesis [18]. Third, endothelial-mesenchymal transition (EndMT), a process in which ECs acquire myofibroblast-like properties, is one of the main mechanisms of atherogenesis that increases endothelial dysfunction [19]. Furthermore, increasing levels of apoptosis and autophagy in ECs can also influence the development of atherosclerosis. The activation of apoptosis [20] and pyroptosis [21] in ECs can increase the levels of inflammatory factors, such as ROS and caspase-1, and lead to vessel wall inflammation, which may be involved in atherogenesis. On the other hand, endothelial autophagy may prolong the survival of ECs by inhibiting endothelial apoptosis and has antiatherogenic effects [22]. Excessive autophagy in ECs can promote atherosclerotic plaque destabilization, which leads to accelerated atherogenesis [23]. Overall, multiple pathways work together to confer a high risk of endothelial dysfunction and atherosclerosis. Therefore, improving endothelial function is critical in the treatment and prognosis of atherosclerosis. Endothelial activity regulated by salidroside are summarized in Table 1.

**Table 1.** **Endothelial activity regulated by salidroside**.

Cell activity	Inflammatory factors or receptors	Possible targeting pathways by SAL	Effect
Endothelial oxidative stress	ROS	cAMP/PKA/RhoA	Downregulate
	IL-1β, IL-6, TNF-α	AMPK/NF-κB/NLRP3	Downregulate
	ROS, MDA	AMPK/SIRT1	Downregulate
	SOD, CAT	Nrf2	Upregulate
Endothelium-dependent contraction	NO	AMPK/PI3K/Akt/eNOS	Upregulate
	Nox2, ROS	—	Downregulate
Endothelial-mesenchymal transition	NO	KLF4/eNOS	Downregulate
Endothelial apoptosis	Bcl-xL	miR-133a	Upregulate
	Caspase-3	—	Downregulate
Endothelial pyroptosis	Caspase-1, IL-1β	—	Downregulate
Endothelial autophagy	LC3-II/ LC3-I	AMPK-mTOR	Upregulate
	—	SIRT1-FoxO1	Upregulate

ROS, Reactive oxygen species; cAMP, Cyclic adenosine monophosphate; PKA, Protein kinase A; RhoA, Ras homolog gene family member A; IL-1β, Interleukin-1β; IL-6, Interleukin-6; TNF-α, Tumor necrosis factor-α; AMPK, AMP-activated protein kinase; SIRT3, Sirtuin 3; NF-κB, Noncanonical nuclear factor-κB; NLRP3, pyrin domain-containing protein 3; SIRT1, Sirtuin-1; MDA, Malondialdehyde; SOD, superoxide dismutase; CAT, catalase; Nrf2, Nuclear factor-erythroid 2-related factor 2; NO, Nitric oxide; PI3K, Phosphatidylinositol-3-kinase; Akt, Protein kinase B; eNOS, Endothelial nitric oxide synthase; Nox2, NADPH oxidases 2; KLF4, Kruppel-like factor 4; Bcl-xL, B-cell lymphoma-extra-large; miR-133a, MicroRNA-133a; LC3-II, Light chain 3-II; LC3-I, Light chain 3-I; mTOR, Mammalian target of rapamycin; FoxO1, Forkhead box O1.

### 2.1 Effects of Salidroside on Endothelial Oxidative Stress

A major cause of endothelial dysfunction is oxidative stress. Salidroside plays a crucial role in downregulating endothelial oxidative stress not only by decreasing the level of proinflammatory factors but also by increasing the level of anti-inflammatory factors. First, ROS have been shown to enhance oxidative stress in ECs [24], which plays an important role in the signaling pathways associated with endothelial dysfunction [25]. Li *et al*. [26] found that the endothelial barrier in intermittent hypoxia (IH)-induced human coronary vein endothelial cell was damaged by ROS, and salidroside (10 μM or 100 μM, 2 h) pretreatment could inhibit ROS overproduction via the cyclic adenosine monophosphate (cAMP)/protein kinase A (PKA)/Ras homolog gene family member A (RhoA) signaling pathway; thus, endothelial barrier function was preserved. Second, noncanonical nuclear factor-κB (NF-κB) plays an important role in endothelial inflammatory responses [27]. It was reported that salidroside (50 μM or 100 μM, 24 h) exerted a protective effect on ECs by activating adenosine monophosphate-activated protein kinase (AMPK) phosphorylation and inhibiting the NF-κB p65/NACHT, LRR, and pyrin domain-containing protein 3 (NLRP3) signaling pathway. In this way, salidroside (0.1 μM, 1 μM or 10 μM, 1 h) can downregulate the levels of proinflammatory factors, such as interleukin-6 (IL-6), interleukin-1β (IL-1β), and tumor necrosis factor-α (TNF-α) [28]. Third, Zhao *et al*. [29] found that salidroside can activate the AMPK/sirtuin-1 (SIRT1) pathway, which inhibits the level of MDA, an oxidative stress index, in Ox-LDL-treated vein endothelial cells (HUVECs). Finally, superoxide dismutase (SOD) and catalase (CAT), which are key antioxidant enzymes that protect against ROS, can also be influenced by salidroside. Zhu and others [30] investigated the effects of different concentrations of salidroside (0.1 μM, 1 μM or 10 μM, 24 h) on the activities of SOD and CAT in hydrogen peroxide ($H_{2}$$O_{2}$)-treated HUVECs. The results showed that salidroside significantly increased cellular SOD and CAT levels, and the antioxidant effect was not proportional to the concentration of salidroside. In addition, this effect might be mediated by activating the nuclear factor-erythroid 2-related factor 2 (Nrf2) signaling pathway.

### 2.2 Effects of Salidroside on Endothelium-Dependent Contraction

Endothelium-dependent contraction (EDC) is also associated with endothelial dysfunction. EDC leads to vasospasm, which may exacerbate ischemia and hypoxia at the beginning of atherogenesis [31]. It is well documented that increased eNOS phosphorylation and expression can enhance NO production to improve endothelial function [32]. Xing *et al*. [33] administered different salidroside (1 μM or 10 μM, 30 min) concentrations to cultured HUVECs. They found that salidroside could increase the adenosine monophosphate (AMP)/adenosine triphosphate (ATP) ratio, which can regulate the activity of AMPK by sequentially depolarizing mitochondria. Thus, salidroside can upregulate NO production by activating the AMPK/protein kinase B (Akt)/eNOS pathway, which can suppress EDC. In another study [34], researchers found that salidroside (100 μM or 300 μM, 1 h) partially ameliorated EDC caused by homocysteine (Hcy) in rat aortic ECs. The researchers measured ROS generation and the expression of NADPH oxidases 2 (Nox2), an oxidase subunit of nicotinamide adenine dinucleotide phosphate (NADPH), and concluded that salidroside could improve NO bioavailability to ameliorate EDC by decreasing the levels of ROS and Nox2.

### 2.3 Effects of Salidroside on Endothelial‑Mesenchymal Transition

EndMT can be exacerbated by inflammation, hypoxia, and oxidative stress in the endothelium through the activation of TGF-β signaling [19]. EndMT increases vascular permeability and disrupts endothelial barrier function [35]. Therefore, LDL can easily accumulate under the vascular endothelium, inducing the formation of atherosclerotic plaques [36]. Moreover, EndMT-derived fibroblast-like cells are associated with plaque instability [37], which exacerbates the progression of atherosclerosis. Some studies have reported that reducing the activation of Krüppel-like factor 4 (KLF4) can inhibit EndMT [38, 39]. Decreasing eNOS activity and phosphorylation results in low NO production and can suppress EndMT [40]. Huang *et al*. [41] showed that salidroside (10 μmol/L or 50 μmol/L, 2 h) could improve the eNOS/NO signaling axis in Hcy-induced EndMT while downregulating the expression levels of KLF4. Therefore, they concluded that salidroside could inhibit EndMT through the KLF4/eNOS signaling pathway [41].

### 2.4 Effects of Salidroside on EC Death

Various forms of endothelial death, such as apoptosis [42], pyroptosis [43], and autophagy [44], can influence the development and progression of atherosclerosis. First, salidroside upregulates the expression of B-cell lymphoma-extra large (Bcl-xL), an antiapoptotic protein, and inhibits Ox-LDL-induced EC apoptosis [45]. Zhang *et al*. [46] used human coronary artery endothelial cell (HCAECs) to analyze the effect of salidroside (100 μM, 24 h) on EC apoptosis. They found that salidroside-mediated inhibition of apoptosis involved the upregulation of microRNA-133a (miR-133) expression because Bcl-xL expression decreased when miR-133a was knocked down in ECs. This result shows that salidroside may inhibit EC apoptosis by upregulating miR-133a expression. Furthermore, Tan and other researchers [47] demonstrated that salidroside (0.1, 1, 10 μg/mL, 2 h) could protect hypoxia-induced ECs from apoptosis by inhibiting the activation of caspase-3, which is known to be a typical marker of cell apoptosis [48]. Moreover, Xing *et al*. [49] found that salidroside (1 μM or 10 μM, 12 h) could suppress lipopolysaccharide (LPS)-induced EC pyroptosis by impairing caspase-1 activation and decreasing IL-1β release. It is well known that the light chain 3-II (LC3-II)/LC3-I ratio is related to the level of autophagy [50]. According to Zheng *et al*. [51], salidroside (100 μM, 12 h) could exert antiapoptotic effects by increasing autophagy. The salidroside pretreatment group exhibited a higher LC3-II/LC3-I ratio than the $H_{2}$$O_{2}$ treatment group. Moreover, the researchers also demonstrated that salidroside (100 μM, 2 h) could markedly increase AMPK phosphorylation but impair mammalian target of rapamycin (mTOR) phosphorylation. These results suggested that salidroside could protect ECs against autophagy by activating the AMPK-mTOR pathway. Finally, Zhu and others [52] reported that salidroside could increase autophagy through the SIRT1-Forkhead box O1 (FOXO1) axis. In this way, salidroside can decrease oxidative stress in HUVECs.

Overall, salidroside can improve endothelial function in many ways, such as through anti-inflammatory effects, increasing the production of NO, inhibiting EndMT, and regulating EC death. These findings support the clinical importance of salidroside.

## 3. Effects of Salidroside on Macrophages

Macrophages play a critical role in the initiation and progression of atherosclerosis. In the early stage of atherosclerosis, macrophages can be recruited to the lesioned arterial wall by proinflammatory cytokines [53]. Macrophage activation is an essential event in early atherosclerosis. Wang and colleagues [54] found that salidroside (50, 100 or 50 μg/L, 24 h) could decrease proinflammatory cytokines, which are released by activated macrophages, by inhibiting the mitogen-activated protein kinase (MAPK)/NF-κB signaling pathway. Second, macrophages can sense and take up lipid particles and transform into foam cells through the upregulation of scavenger receptors, such as CD36, scavenger receptor A1 (SR-A1), and lectin-like Ox-LDL receptor-1 (LOX-1) [7]. In advanced atherosclerosis, macrophage proliferation is another crucial mechanism that increases the progression of plaques [55]. Ni and other scholars [56] discovered that salidroside (0.1, 1, 10 μM, 5 h) could attenuate the expression of LOX1 and lower lipid accumulation in Ox-LDL-treated THP1 cells. These beneficial effects were partly mediated by activating the MAPK/Akt signaling pathway.

Finally, studies have shown that different macrophage phenotypes play different roles in atherosclerosis. It is widely known that M1 macrophages play a proinflammatory role in atherosclerosis, while M2 macrophages play a preventive role [57, 58, 59]. Li *et al*. [60] discovered that salidroside (25~100 μg/mL, 12 h) could suppress the activation of M1 macrophages by downregulating the Notch1-HES1 signaling pathway. In this way, salidroside could also attenuate the release of TNF-α, IL-6, IL-1β, and monocyte chemoattractant protein 1 (MCP-1) by impairing proinflammatory M1 activation. In addition, arachidonic acid has been reported to be involved in inhibiting M2 polarization [61], while STAT1 and NF-κB are two important transcription factors that can increase the activation of M1 cells [62]. Liu *et al*. [63] found that salidroside could suppress macrophage polarization. The researchers established a gouty arthritis rat model to observe the effects of salidroside (80 mg/kg, i.g., 6 d) on macrophage phenotypic switching. Salidroside could reprogram COX-2-, 5-LOX-, and CYP4A-mediated arachidonic acid metabolism through STAT1/NF-κB signaling. Therefore, salidroside can attenuate the activation of THP-1-cell-derived macrophages and decrease the release of inflammatory factors.

## 4. Effects of Salidroside on VSMCs

VSMCs are one of the main cell types in the blood vessel wall. Increased VSMC proliferation can induce pathological intimal thickening, which can induce the progression of atherosclerosis [64]. Some studies have shown that VSMCs switch from a contractile to synthetic phenotype, and these cell possess highly proliferative and migratory capacities, which may impair plaque stability [64, 65]. In addition, atherosclerotic plaque stability is negatively associated with increased VSMC apoptosis [66]. Whether salidroside can inhibit the switching of VSMCs is still unclear and needs further examination. The studies which have focused on the beneficial effects of salidroside on inhibiting VSMC proliferation and apoptosis are as follows.

Zhuang and other researchers [67] investigated the protective effect of salidroside (0.3 and 0.5 mM, 24 h) on VSMCs under high glucose stimulation. The results showed that salidroside could decrease the proliferation of VSMCs not only by downregulating the activation of NADPH and reducing the level of ROS but also by inhibiting mitochondrial fission through the downregulation of dynamin-related protein (Drp1) and mitofusin 2 (Mfn2). Moreover, salidroside (100 μM, 1 h) has been reported to inhibit the proliferation of VSMCs by blocking the AKT/glycogen synthase kinase 3 β (GSK3β) signaling pathway [68]. Hypoxia/reperfusion (H/R) can increase the expression of inflammatory molecules and exacerbate oxidative stress [69], which may lead to the cardiotoxic effects of VSMCs. Xu *et al*. [70] examined the viability, caspase-3 activity and apoptosis rate of VSMCs to determine the potential mechanism by which salidroside (100, 200 or 400 μM, 30 min) antagonizes H/R-induced cell apoptosis. The results confirmed that salidroside could reverse H/R-induced cell apoptosis by enhancing the activation of the SIRT1/FoxO3α pathway. Thus, salidroside can suppress the proliferation of VSMCs by downregulating Drp1, Mfn2 and oxidative stress, as well as by inhibiting the AKT/GSK3β signaling pathway. In addition, salidroside can reduce VSMC apoptosis by enhancing the activation of the SIRT1/FoxO3α pathway.

## 5. Effects of Salidroside on Platelets

Platelet activation leads to adhesion, aggregation, and thrombosis, playing a significant role in atherosclerosis [71]. Recently, antiplatelet therapies, such as aspirin, clopidogrel, and ticagrelor, have been shown to play a significant role in reducing clinical atherothrombotic events among high-risk patients [72]. Salidroside, which is a botanical medicine, has also been demonstrated to produce beneficial effects on inhibiting platelets.

Wei *et al*. [73] demonstrated that salidroside (5, 10 and 20 μM, 1 h) could inhibit thrombin- or C-reactive protein (CRP)-induced human platelet aggregation, and this finding was consistent with a study in mouse platelets. Moreover, the researchers found that salidroside could not only attenuate platelet aggregation but also inhibit hemostasis and arterial thrombus formation *in vivo* through AKT/GSK3β signaling. Although more research is needed to empirically determine the mechanism by which salidroside affects platelets, these results provide new ideas for salidroside as a novel antiplatelet therapy.

## 6. Effects of Salidroside on Lipid Metabolism

An aberrant lipid profile, including increased total cholesterol (TC), triglyceride (TG), and LDL-C and decreased high-density lipoprotein cholesterol (HDL-C), is associated with an increased risk of atherosclerosis [74]. Therefore, lipid lowering is regarded as the key treatment in the primary and secondary prevention of atherosclerosis. Currently, statins, and proprotein convertase subtilisin/Kexin type 9 (PCSK9) inhibitors, and icosapent ethyl (IPE), which are essential lipid-lowering therapies, play vital roles in controlling atherosclerosis [75]. The studies which have shown that salidroside may also lower lipid levels are as follows.

First, some animal studies have suggested that salidroside (100 mg⋅${\mathrm{kg}}^{-1}$⋅${\mathrm{day}}^{-1}$, peros (p.o.), 8 weeks) could induce abnormal lipid accumulation by stimulating the phosphorylation of AMPK in hepatocytes [76, 77, 78]. Second, Zhang and colleagues [79] found that although salidroside (50 mg⋅${\mathrm{kg}}^{-1}$⋅${\mathrm{day}}^{-1}$, p.o., 8 weeks) could not decrease the weights of mice fed a high-fat diet (HFD), it could lower the levels of TC and TG and increase HDL-C. Thus, the plaque area was significantly decreased in response to salidroside. These results show that salidroside can decrease atherosclerotic plaque formation by ameliorating lipid imbalances. In addition, Wen *et al*. [80] used salidroside (8 mg/kg and 6 mg⋅${\mathrm{kg}}^{-1}$⋅${\mathrm{day}}^{-1}$, intraperitoneal injection, 16 weeks) to treat an apoE^-/-^ mouse model, which developed atherosclerotic lesions similar to those in humans. They reached the same conclusion. Third, some researchers used HFD-fed mice and observed whether salidroside could reduce serum lipids. The researchers analyzed body weight, abdominal fat and serum levels of TC, HDL-C, LDL-C, and TG. The results showed that salidroside (25 and 50 mg⋅${\mathrm{kg}}^{-1}$⋅${\mathrm{day}}^{-1}$, p.o., 8 weeks) could inhibit the serum levels of TC and LDL-C but had no significant effects on TG or HDL-C [33]. These results suggested that salidroside could not only significantly protect against the increase in atherosclerotic plaques but also alleviate abnormal TC accumulation. Finally, salidroside (25 and 50 mg⋅${\mathrm{kg}}^{-1}$⋅${\mathrm{day}}^{-1}$, p.o., 12 weeks) significantly inhibited the insulin-induced gene 1 (INSIG1)-sterol regulatory element-binding protein (SREBP) pathway and could suppress the gene expression of ATP citrate lyase to inhibit de novo lipogenesis and cholesterol biosynthesis [81]. In addition, as a key transcriptional regulator of lipogenesis, SREBP-1 promotes lipid accumulation [82]. Zhang *et al*. [83] demonstrated that the miR-370 inhibitor could inhibit the expression of SREBP-1c by 36%. With a further study, they found that salidroside plays an important role in the downregulation of miR-370. This finding suggests that salidroside (40, 80 and 160 mg⋅${\mathrm{kg}}^{-1}$⋅${\mathrm{day}}^{-1}$, p.o., 4 weeks) may be a potential target for the treatment of lipid metabolism. In summary, salidroside may be a new therapeutic drug for balancing the levels of serum lipids and alleviating the development of plaque areas.

## 7. Effects of Salidroside on the Gut Microbiota

Recent research has highlighted the significant role of the gut microbiota in CVD [84], especially in atherosclerosis. On the one hand, some studies have shown that gut dysbiosis plays an important role in atherosclerosis [85]. On the other hand, increasing intestinal permeability and disruption of the intestinal barrier can lead to bacterial translocation, which may release LPS, trimethylamine (TMA) and trimethylamine-N-oxide (TMAO) into the circulation. These gut microbiota-derived products can not only induce systemic inflammation but are also connected with atherosclerosis [86]. In other words, dysregulation of the gut microbiota leads to low-grade chronic inflammation, which can accelerate atherosclerotic progression [87]. Zhu *et al*. [52] collected fecal samples from 218 individuals with atherosclerotic cardiovascular disease (ASCVD) and compared the composition of the gut microbiota with the samples from healthy controls. They discovered that ASCVD patients had a higher level of Streptococcus and Escherichia [88]. Moreover, scholars from Japan reported that Lactobacillales was increased in CAD patients, while Bacteroidetes was decreased [89]. In addition, changes in the gut microbiota and gut permeability can increase IL-6, TNF-α [90], dyslipidemia, and ectopic fat deposition [91].

Several studies have reported the protective effect of salidroside on the gut microbiome. First, Xie and other scholars [92] observed that salidroside (50 mg⋅${\mathrm{kg}}^{-1}$⋅${\mathrm{day}}^{-1}$, i.g., 12 weeks) could increase the levels of the proteins Zona occludens 1 (ZO-1) and occludin, which could strengthen the integrity and tight junctions of the intestine [93]. Thus, salidroside can restore intestinal barrier integrity and intestinal permeability, which may reduce the accumulation of microbial products in the periphery and reduce chronic inflammation. Moreover, salidroside can regulate the gut microbiota in mice, especially the levels of Lactobacillus and Alloprevotella spp. Second, Li *et al*. [94] analyzed the composition of the gut microbiota in salidroside (20 mg⋅${\mathrm{kg}}^{-1}$⋅${\mathrm{day}}^{-1}$, i.g., 4 weeks) -treated and HFD-fed mice. They observed that the relative levels of Lactobacillus and Alloprevotella spp. in the intestinal tract were suppressed by salidroside. Furthermore, Chen *et al*. [95] discovered that salidroside (10, 20 and 40 μM, 2 h) could protect against LPS-induced injury. They observed that salidroside could suppress LPS-induced ROS production through the PI3K/Akt/mTOR pathway. Salidroside has advantages in preserving the intestinal barrier, but the underlying mechanism still requires more research.

## 8. Conclusions and Future Perspectives

This review provides a modern scientific perspective to further understanding the molecular mechanism of salidroside attenuating atherosclerosis and supply new ideas for atherosclerosis management.

Based on the present studies, salidroside affects atherosclerosis through multiple signaling pathways and related mechanisms. Salidroside protects against atherosclerosis through multiple targets and multiple pathways. (1) Salidroside ameliorates endothelial dysfunction through anti-inflammatory effects, increasing the production of NO, inhibiting EndMT, and regulating the death of ECs. (2) Salidroside suppresses macrophage activation by inhibiting the MAPK/NF-κB signaling pathway. In addition, it can also reduce foam cell formation by activating the Akt/MAPK pathway. Furthermore, macrophage polarization can be suppressed by salidroside via STAT1/NF-κB signaling. (3) Salidroside suppresses the proliferation of VSMCs by inhibiting the AKT/GSK3β signaling pathway or enhancing the activation of the SIRT1/FoxO3α pathway. (4) Salidroside can ameliorate lipid imbalance. There may be several underlying mechanisms. Salidroside decreases the INSIG1-SREBP pathway and downregulates the expression of miR-370 to adjust lipid metabolism. (5) Salidroside can induce platelet aggregation by inhibiting thrombin or CRP. In addition, salidroside can suppress thrombus formation *in vivo* through AKT/GSK3β signaling. (6) Salidroside can strengthen the intestinal barrier and improve intestinal permeability by increasing ZO-1 and occludin protein levels. Additionally, salidroside can regulate the gut microbiota and reduce ROS via the PI3K/Akt/mTOR pathway, which can improve the gut microenvironment.

In conclusion, these findings suggest that salidroside may be a promising drug for preventing and treating atherosclerosis. At present, the anti-atherosclerotic signaling pathways and targets of salidroside are not comprehensively understood, and few animal studies have been conducted. Besides, its clinical application has progressed slowly and some details remain unknown, and the best optimum dose is not determined. Some studies were restricted to a single model, and toxicity issues were not included. Therefore, more studies, especially clinical trials, are needed to further confirm the therapeutic effects and molecular mechanisms of salidroside.
